# Blood-brain barrier disruption induced by diagnostic ultrasound combined with microbubbles in mice

**DOI:** 10.18632/oncotarget.23527

**Published:** 2017-12-21

**Authors:** Bingxia Zhao, Yihan Chen, Jinfeng Liu, Li Zhang, Jing Wang, Yali Yang, Qing Lv, Mingxing Xie

**Affiliations:** ^1^ Department of Ultrasound, Union Hospital, Tongji Medical College, Huazhong University of Science and Technology, Wuhan 430022, China; ^2^ Hubei Key Laboratory of Molecular Imaging, Union Hospital, Tongji Medical College, Huazhong University of Science and Technology, Wuhan 430022, China

**Keywords:** blood-brain barrier, diagnostic ultrasound, microbubble, drug delivery

## Abstract

**Objective:**

To investigate the effects of the microbubble (MB) dose, mechanism index (MI) and sonication duration on blood-brain barrier (BBB) disruption induced by diagnostic ultrasound combined with MBs as well as to investigate the potential molecular mechanism.

**Results:**

The extent of BBB disruption increased with MB dose, MI and sonication duration. A relatively larger extent of BBB disruption associated with minimal tissue damage was achieved by an appropriate MB dose and ultrasound exposure parameters with diagnostic ultrasound. Decreased expression of ZO-1, occludin and claudin-5 were correlated with disruption of the BBB, as confirmed by paracellular passage of the tracer lanthanum nitrate into the brain parenchyma after BBB disruption.

**Conclusions:**

These findings indicated that this technique is a promising tool for promoting brain delivery of diagnostic and therapeutic agents in the diagnosis and treatment of brain diseases.

**Methods:**

The extent of BBB disruption was qualitatively assessed by Evans blue (EB) staining and quantitatively analyzed by an EB extravasation measurement. A histological examination was performed to evaluate tissue damage. Expression of tight junction (TJ) related proteins ZO-1, occludin and claudin-5 was determined by western blotting analysis and immunohistofluorescence. Transmission electron microscopy was performed to observe ultrastructure changes of TJs after BBB disruption.

## INTRODUCTION

Brain diseases, including neurodegenerative diseases and brain tumors, are currently still presenting enormous challenges for clinicians [1, 2]. Although many new diagnostic and therapeutic drugs have been developed, drug delivery to the brain is severely limited due to the existence of the blood-brain barrier (BBB). The BBB serves as a physical and physiological barrier that prevents all large molecule drugs and more than 98% of small molecule drugs (>400 Da) from entering into the central nervous system (CNS) [3]. The BBB is composed of specialized endothelial cells (ECs) linked to each other by tight junctions (TJs), a basement membrane, astrocytic foot processes, perivascular pericytes, macrophages and microglial cells [4]. TJs, which consist of transmembrane proteins occludin and claudins, submembranous zonula occludens proteins and the cytoskeleton of ECs, are primarily responsible for the characteristics of the BBB [5].

To deliver diagnostic and therapeutic agents into the CNS, various strategies have been investigated to increase the permeability of the BBB such as converting water-soluble small molecule drugs into lipid-soluble ones or connecting them to carriers that can cross the BBB [6, 7], internal carotid artery injection of hypertonic solutions [8], intra-arterial infusion of inflammatory mediators such as bradykinin or its analogue RMP-7 [9], direct intracerebral injection/infusion [10], convection-enhanced delivery [11] and intranasal administration [12]. However, all of these methods are limited either by invasive; off-target effects; poor delivery efficiency; or the risk of surgical complications, including vascular lesions, neurological damage and infection. Therefore, examining noninvasive and effective ways to facilitate drug delivery into the brain is becoming increasingly important.

Previous studies have demonstrated that focused ultrasound (FUS) combined with system administrated microbubbles (MBs) can noninvasively, locally and temporarily disrupt the BBB in rodents [13], rabbits [14], pigs [15] and non-human primates [16]. Therefore, diagnostic and therapeutic agents can be locally delivered into the brain during a time window of BBB disruption, which typically lasts several hours [17–19]. Until now, using FUS combined with MBs, various substances, such as chemotherapeutic agents [20], molecular imaging agents [21], antibodies [22], neurotrophic factors [23], genes [1], nanoparticles [24], and even cells [25], have been delivered into targeted brain regions for brain tumor and neurodegenerative disease imaging and treatment.

Nevertheless, focus spots of FUS transducers are generally 2–3 mm [13, 25]. With regard to the diseases with unclear lesions or lesions extensively distributed in the brain, larger BBB disruption is necessary and FUS is limited. Unfocused diagnostic ultrasound combined with MBs can open the entire brain, and may serve as a promising method to broadly deliver diagnostic and therapeutic agents [26]. Meanwhile, diagnostic ultrasound is capable of providing real-time image guidance without the aid of additional devices. Moreover, diagnostic ultrasound systems are more accessible to biomedical researchers. Despite the above advantages, few studies applied diagnostic ultrasound to BBB disruption [27–29]. What's more, studies on the influence of various experimental parameters were fewer. Therefore, how to make the balance between safety and efficacy is still one of urgent problems of applying diagnostic ultrasound to BBB disruption.

In this study, we investigated the effects of MB dose, mechanical index (MI) and sonication duration on BBB disruption under the flash mode, aiming to determine optimal parameters that could maximize the delivery of drugs into the brain and minimize tissue damage. The duration of BBB disruption under optimal parameters was evaluated. Additionally, we examined the response of TJs in brain microvessels following BBB disruption induced by the combination of diagnostic ultrasound and MBs.

## RESULTS

### Characterization of MBs

**Figure 1:**
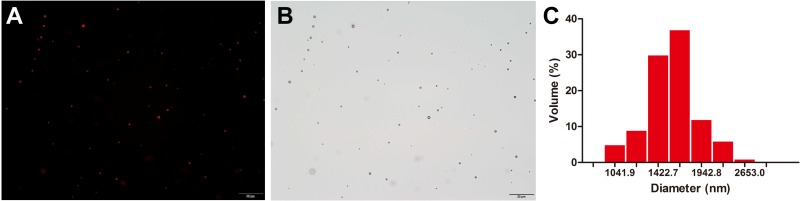
Physical property of MBs (**A**) Fluorescence image. (**B**) Bright field image of MBs. (**C**) Size distribution of MBs measured by a dynamic light-scattering system. Scale bar = 20 μm.

Figure 1A illustrated the morphology of MBs with a dense layer of 1,1′-dioctadecyl-3,3,3′,3′-tetramethylindocarbocyanine perchlorate (DiI) on the lipid shell in the fluorescence image, which was consistent with that revealed in the bright field image (Figure 1B). MBs displayed a uniform size distribution with a single peak as shown in Figure 1C. Mean size of MBs was 1546.8 ± 114.2 nm with a polydispersity index of 0.161 ± 0.018. Mean concentration of MBs was (3.71 ± 0.17) × 10^9^ MBs/ml.

### Effect of MB dose on BBB disruption

**Figure 2:**
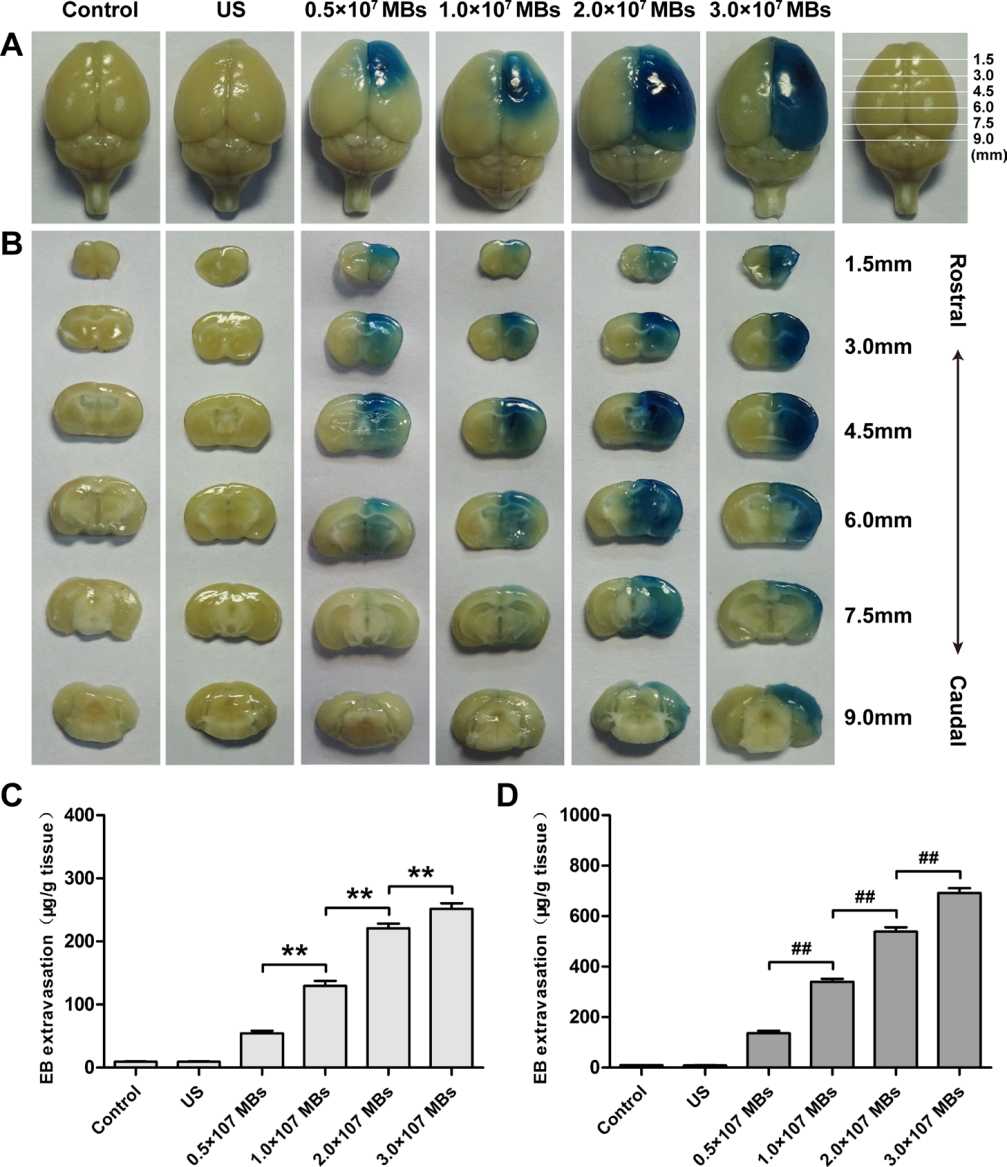
Distribution of EB extravasation in the surface view (**A**) and coronal sections (**B**) of mice brains after treatment of different MB doses at a fixed MI and sonication duration. Relationship between EB extravasation and MB doses in the cortex (**C**) and striatum (**D**) of mice at a fixed MI and sonication duration. Data were presented as the mean ± SEM, *n* = 4. ^**^ and ^##^*P <* 0.01. Control, without sonication or MB injections; US, only sonication and without MB injections.

Next, we investigated the influence of MB dose on BBB disruption in the right hemisphere. Evans Blue (EB) was employed as an indicator for evaluating the permeability of the BBB. Figure 2A and 2B showed the relationship between MB dose and the extent of EB staining in the surface view and coronal sections of the brains. Degree and volume of EB staining in the sonicated right hemisphere increased with MB dose. Groups with an injection of 2.0 × 10^7^ MBs or 3.0 × 10^7^ MBs induced a more profound BBB disruption effect and more intense EB staining in the brain parenchyma than that of 0.5 × 10^7^ MBs or 1.0 × 10^7^ MBs. However, there was no detectable EB staining in the group that only received sonication without injection of MBs as well as in the control group.

Then, quantitative analysis of EB extravasation after different MB dose injections was performed in the cortex and striatum of the right hemisphere. With an increase in MB dose, the amount of EB extravasation increased in both the cortex and striatum (Figure 2C and 2D). In the 2.0 × 10^7^ MBs and 3.0 × 10^7^ MBs groups, the amount of EB extravasation in the cortex and striatum were significantly greater than that of the 0.5 × 10^7^ MBs and 1.0 × 10^7^ MBs groups. Moreover, when MB dose increased from 2.0 × 10^7^ MBs to 3.0 × 10^7^ MBs, EB extravasation in the striatum increased more prominently than in the cortex. Concurrently, EB extravasation in the cortex and striatum of the ultrasound only group was comparable with that in the control group.

**Figure 3:**
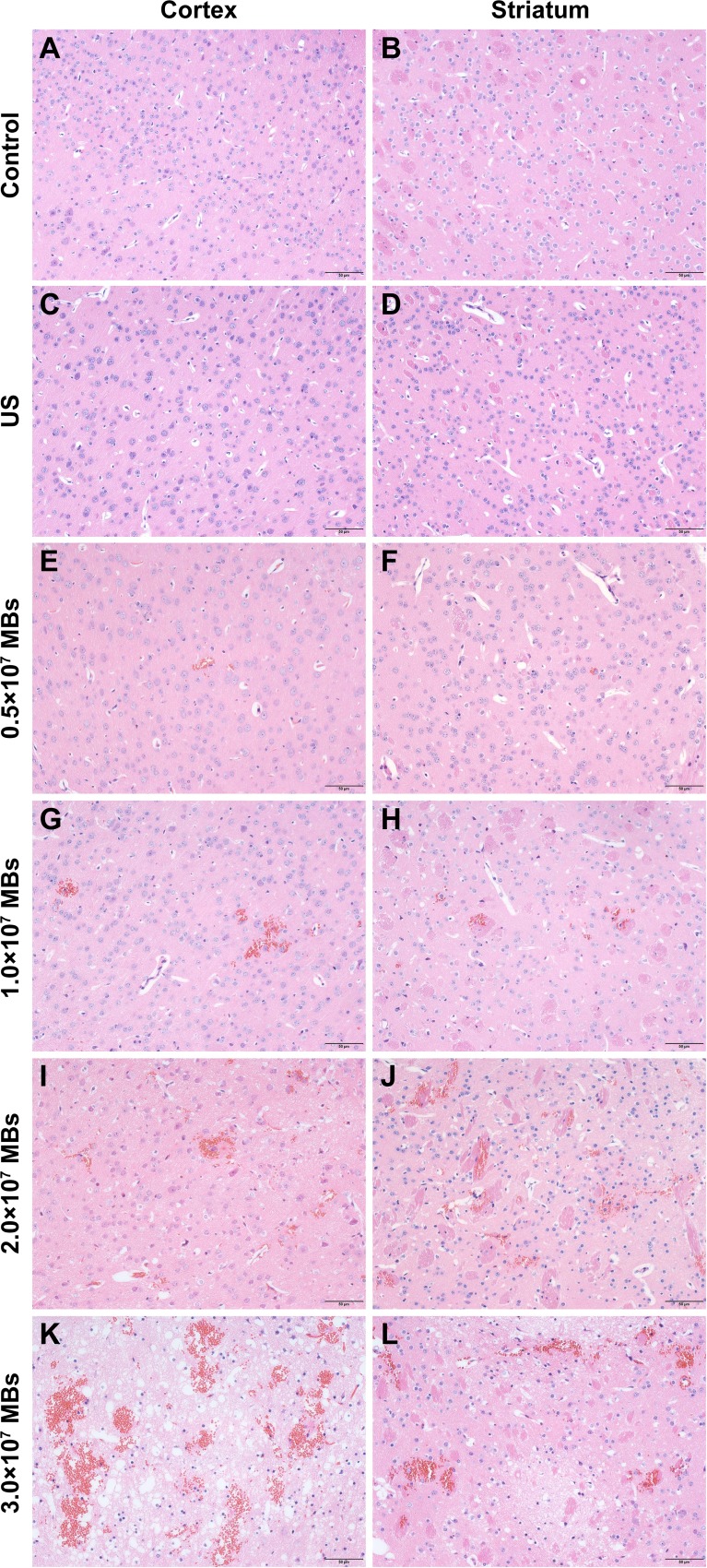
Representative coronal hematoxylin and eosin (H&E) stained sections of the cortex and striatum obtained at different MB doses (**A**, **B**) H&E stained sections of the cortex and striatum of control group. (**C**, **D**) H&E stained sections of the cortex and striatum of US group. (**E**, **F**) H&E stained sections of the cortex and striatum of 0.5 × 10^7^ MBs group. (**G**, **H**) H&E stained sections of the cortex and striatum of 1.0 × 10^7^ MBs group. (**I**, **J**) H&E stained sections of the cortex and striatum of 2.0 × 10^7^ MBs group. (**K**, **L**) H&E stained sections of the cortex and striatum of 3.0 × 10^7^ MBs group. Control, without sonication or MB injections; US, only sonication and without MB injections. Scale bar = 50 μm.

Histological findings of brains obtained from mice treated with different MB doses were shown in Figure 3. As the control group (Figure 3A and 3B), there was no erythrocyte extravasation or tissue damage detected in the ultrasound only group (Figure 3C and 3D). A few scattered erythrocytes to small groups of erythrocyte extravasation were observed at lower MB doses of (0.5–1.0) × 10^7^ MBs without additional tissue damage (Figure 3E–3H). The degree of erythrocyte extravasation increased with MB dose, increasing to 2.0 × 10^7^ MBs, associated with individual dark-stained ischemic or apoptotic neurons and slight vacuolization of the neuropil surrounding impaired vessels (Figure 3I and 3J). The most extensive erythrocyte extravasation along with distinct neuron loss and acute degeneration of the neuropil was detected in the group injected with 3.0 × 10^7^ MBs (Figure 3K and 3L).

### Effect of MI on BBB disruption

**Figure 4:**
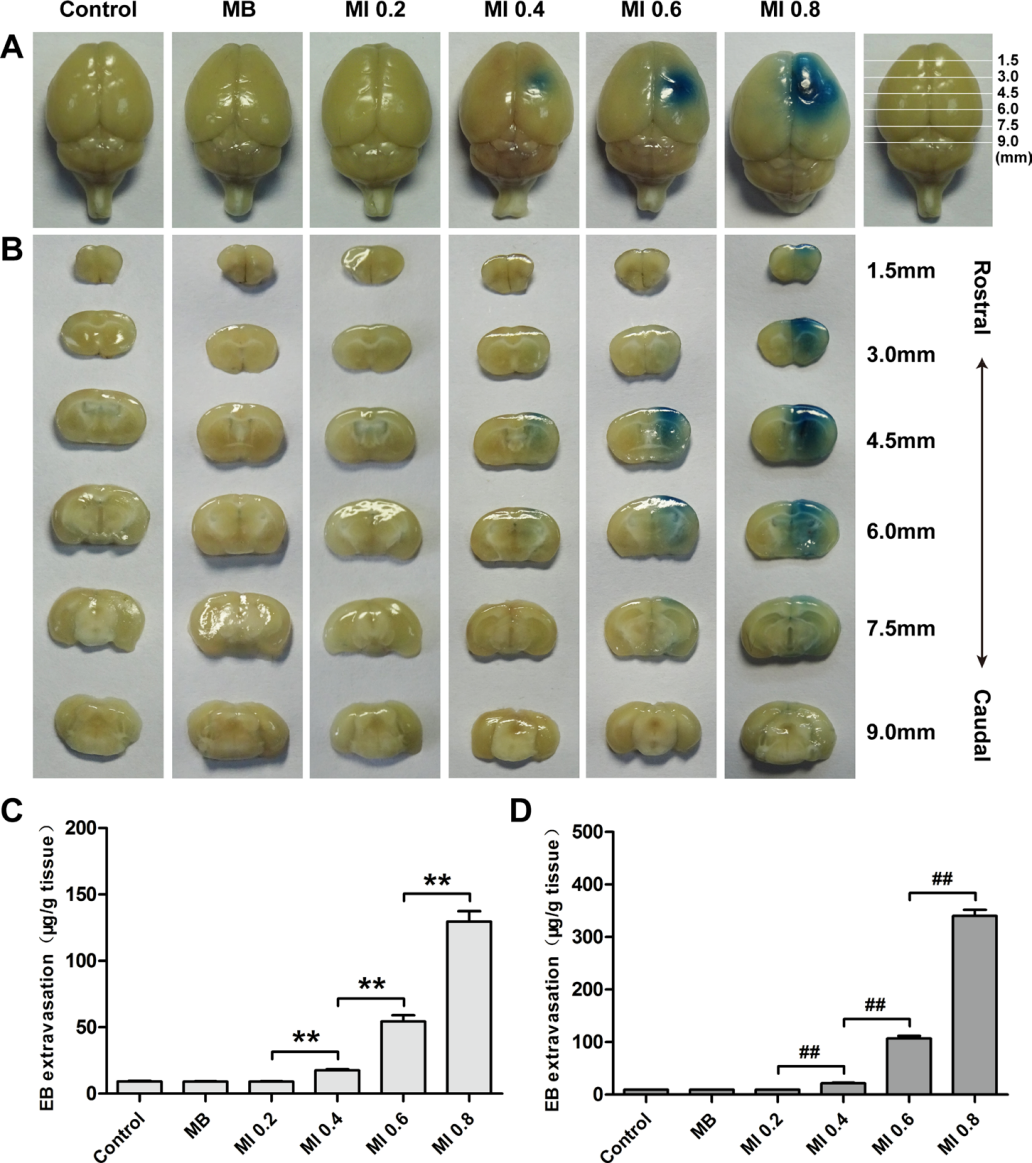
Distribution of EB extravasation in the surface view (**A**) and coronal sections (**B**) of mice brains sonicated with different MIs at a fixed MB dose and sonication duration. Relationship between EB extravasation and MI in the cortex (**C**) and striatum (**D**) of mice at a fixed MB dose and sonication duration. Data were presented as the mean ± SEM, *n* = 4. ^**^ and ^##^*P <* 0.01. Control, without sonication or MB injections; MB, only MB injections and without sonication.

Extent of BBB permeability was enhanced with an increase of ultrasonic MI at a fixed MB dose and sonication duration (Figure 4A and 4B). There was no visible EB staining when a MI of 0.2 was applied. When MI increased to 0.4, slight EB staining could be detected in the surface view and coronal sections of the brains. BBB permeability was significantly enhanced at a MI of 0.6 and 0.8 than at 0.4, particularly at a MI of 0.8.

The relationship between MI and the amount of EB extravasation was shown in Figure 4C and 4D. Until MI reached 0.4, a small amount of EB extravasation was detected in the cortex and striatum. In the groups sonicated with a MI of 0.6 and 0.8, the amount of EB extravasation increased more remarkably than that at a MI of 0.4. Compared with the group with a MI of 0.6, the EB extravasation in the striatum increased nearly 3.19 times in the group sonicated with a MI of 0.8, while the EB extravasation in the cortex only increased 2.38 times.

**Figure 5:**
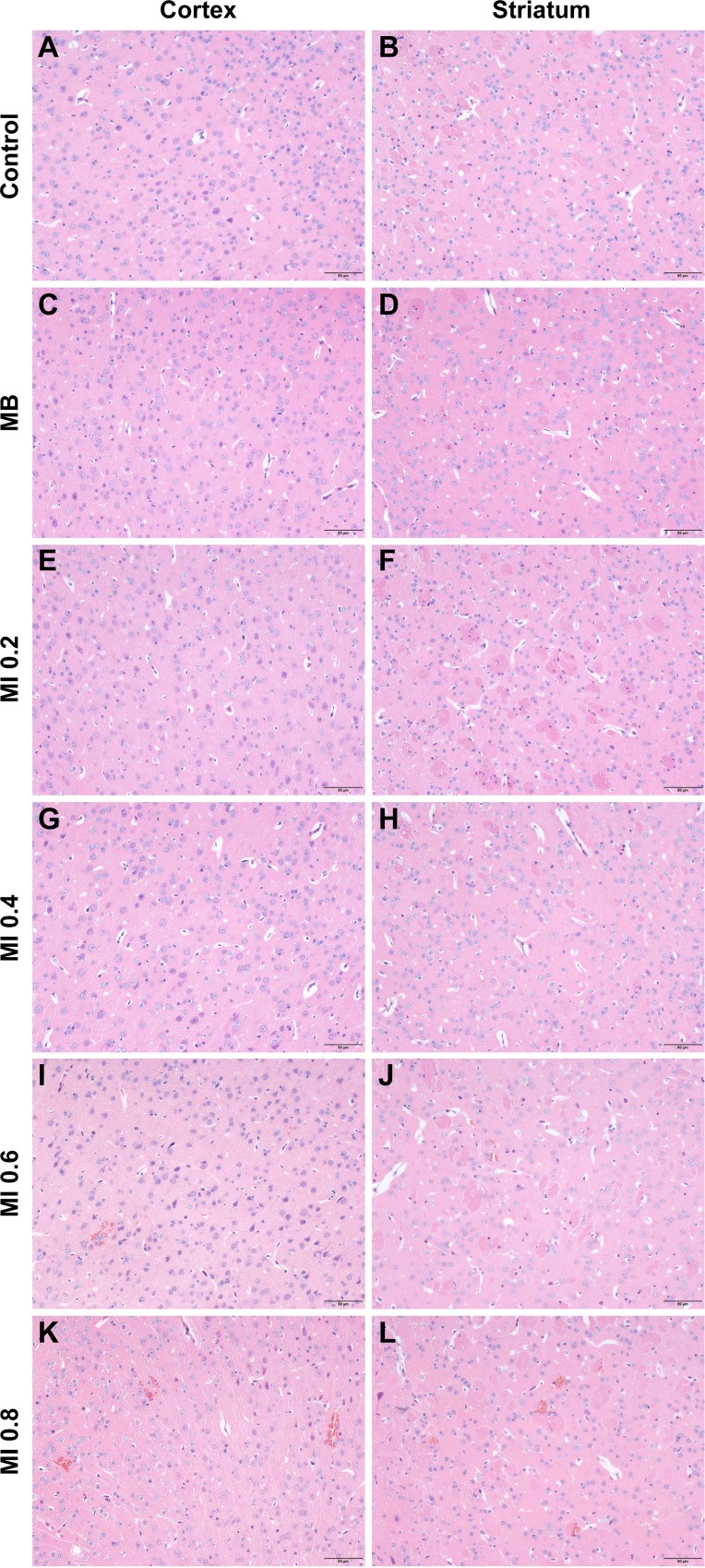
Representative coronal hematoxylin and eosin (H&E) stained sections of the cortex and striatum obtained at different MIs (**A**, **B**) H&E stained sections of the cortex and striatum of control group. (**C**, **D**) H&E stained sections of the cortex and striatum of US group. (**E**, **F**) H&E stained sections of the cortex and striatum of MI 0.2 group. (**G**, **H**) H&E stained sections of the cortex and striatum of MI 0.4 group. (**I**, **J**) H&E stained sections of the cortex and striatum of MI 0.6 group. (**K**, **L**) H&E stained sections of the cortex and striatum of MI 0.8 group. Control, without sonication or MB injections; MB, only MB injections and without sonication. Scale bar = 50 μm.

Histological evaluation did not show any damage to blood vessels or brain tissue when the MI was less than or equal to 0.4 (Figure 5C–5H), just like the control group (Figure 5A and 5B). When the MI increased from 0.6 to 0.8, the only discernable abnormalities were a few scattered erythrocytes and occasionally small groups of erythrocyte extravasation in the cortex and striatum, which indicated a small amount of capillaries were mildly impaired (Figure 5I–5L). It appeared that the increased erythrocyte extravasation was related to the increase of MI.

### Effect of sonication duration on BBB disruption

**Figure 6:**
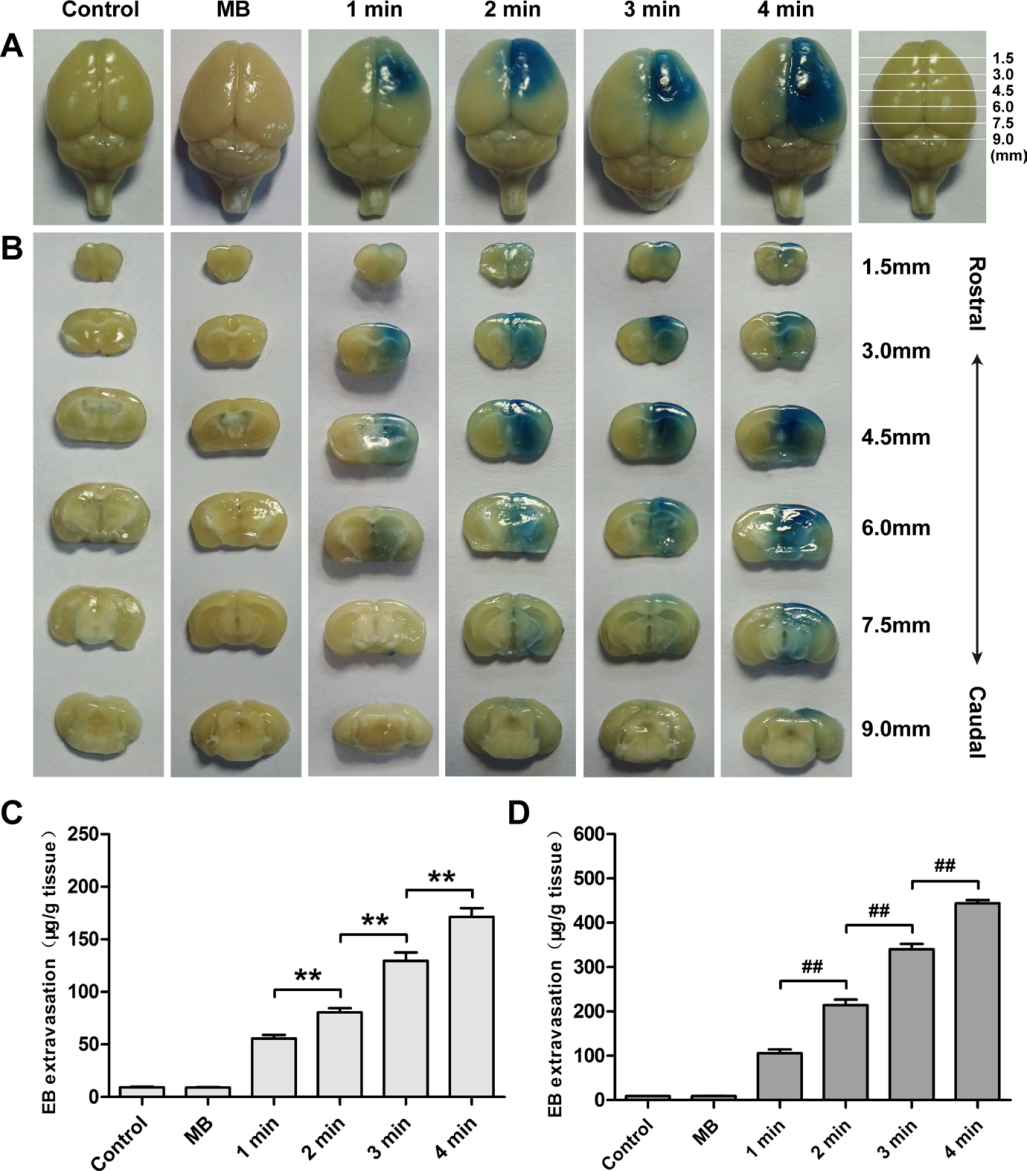
Distribution of EB extravasation in the surface view (**A**) and coronal sections (**B**) of mice brains sonicated with different sonication durations at a fixed MB dose and MI. Relationship between EB extravasation and sonication duration in the cortex (**C**) and striatum (**D**) of mice at a fixed MB dose and MI. Data were presented as the mean ± SEM, *n* = 4. ^**^ and ^##^*P <* 0.01. Control, without sonication or MB injections; MB, only MB injections and without sonication.

BBB permeability was correlated with sonication duration when MB dose and MI were fixed (Figure 6A and 6B). Degree of EB staining increased monotonically with an increase in sonication duration from 1 min to 4 min. It was additionally found that the group with MB injections without sonication did not significantly enhance EB extravasation.

Sonication duration was another important factor to enhance EB extravasation into the brain. EB extravasation in the cortex and striatum additionally increased as a function of sonication duration (Figure 6C and 6D). The amount of EB extravasation in the cortex in the 1 min, 2 min, 3 min and 4 min groups were 6.05, 8.75, 14.05 and 18.58 times higher than that in the control group, respectively. However, the amount of EB extravasation in the striatum for these groups was 11.39, 23.14, 36.73 and 47.94 times higher than that in the control group.

**Figure 7:**
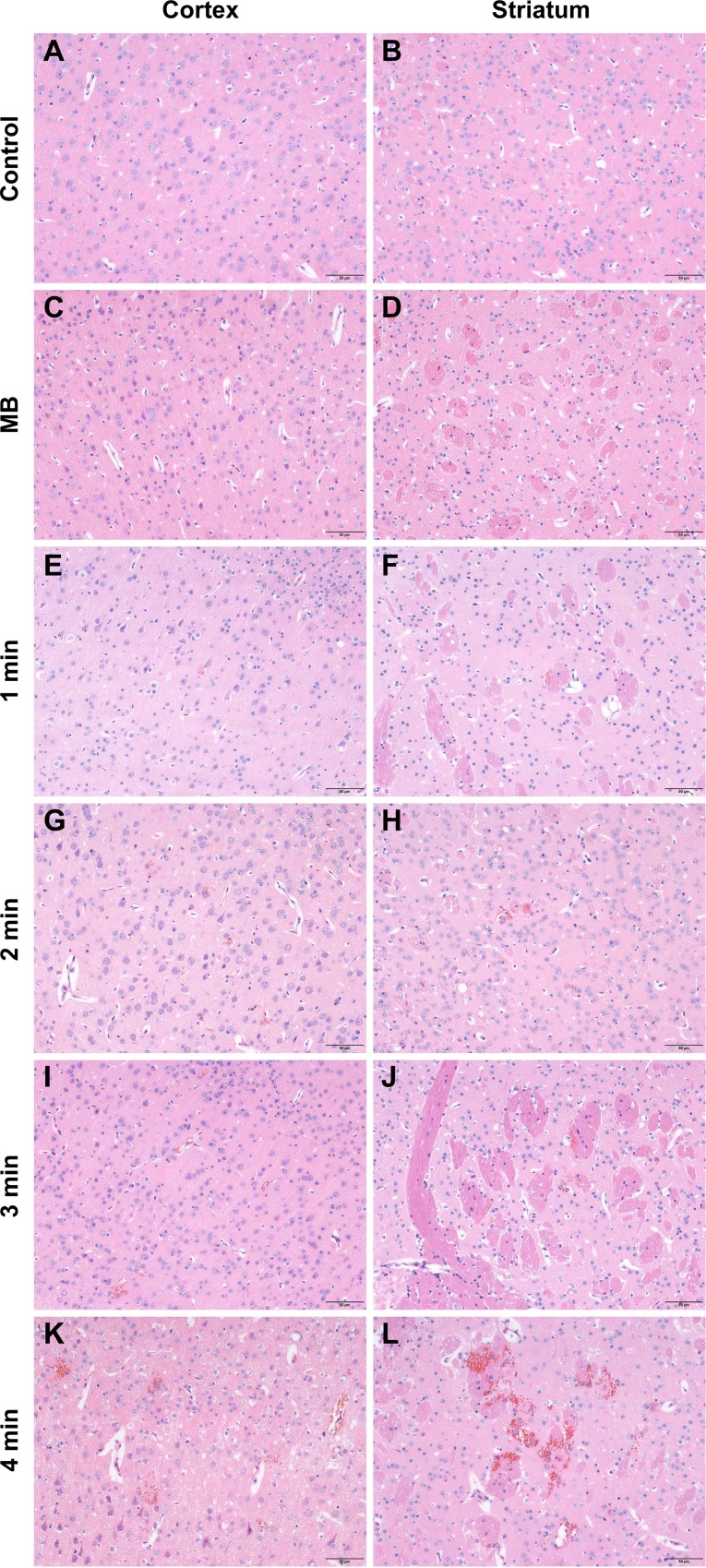
Representative coronal hematoxylin and eosin (H&E) stained sections of the cortex and striatum of mice obtained at different sonication durations (**A**, **B**) H&E stained sections of the cortex and striatum of control group. (**C**, **D**) H&E stained sections of the cortex and striatum of US group. (**E**, **F**) H&E stained sections of the cortex and striatum of 1 min group. (**G**, **H**) H&E stained sections of the cortex and striatum of 2 min group. (**I**, **J**) H&E stained sections of the cortex and striatum of 3 min group. (**K**, **L**) H&E stained sections of the cortex and striatum of 4 min group. Control, without sonication or MB injections; MB, only MB injections and without sonication. Scale bar = 50 μm.

Compared with the control group (Figure 7A and 7B) and the ultrasound only group (Figure 7C and 7D), only a few scattered erythrocyte extravasation was observed in the cortex and striatum after a 1 min sonication duration (Figure 7E and 7F). When sonication duration increased to 3 min, the number and size of erythrocyte extravasation increased. However, no obvious evidence of damage to the parenchyma was observed (Figure 7G–7J). In contrast, a 4 min sonication produced a larger degree of erythrocyte extravasation in conjunction with slight vacuolization of the neuropil, occasionally along with neuron apoptosis (Figure 7K and 7L).

Based on the extent of BBB disruption and histological findings, a combination of a MB dose of 1.0 × 10^7^ MBs, a MI of 0.8 and a sonication duration of 3 min was the relatively appropriate parameter for BBB disruption and was used in the following experiments.

### Duration of BBB disruption

**Figure 8:**
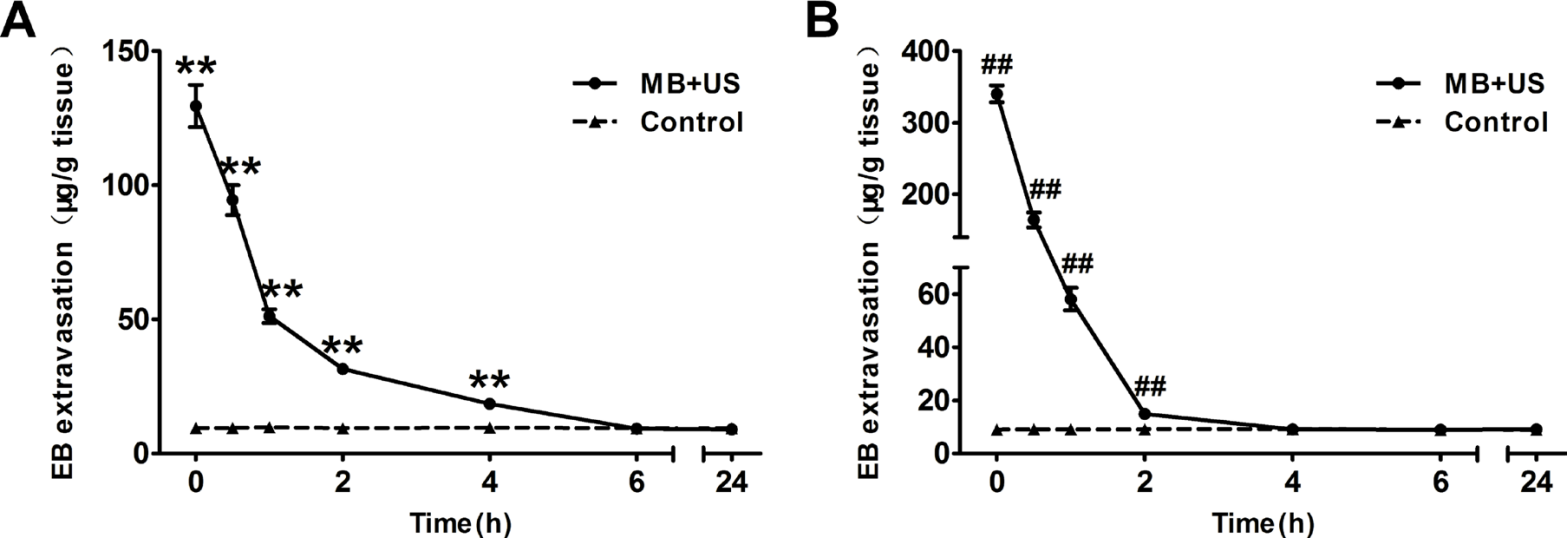
EB extravasation in the cortex (**A**) and striatum (**B**) of mice at each time point after sonication (0 h, 0.5 h, 1 h, 2 h, 4 h, 6 h and 24 h). Data were presented as the mean ± SEM, *n* = 4. ^**^ and ^##^*P <* 0.01. Control, without sonication or MB injections.

To monitor the change of BBB permeability after BBB disruption, EB was intravenously injected at 0 h, 0.5 h, 1 h, 2 h, 4 h, 6 h and 24 h after sonication. As shown in Figure 8A and 8B, the highest EB extravasation was achieved by injecting EB immediately after sonication in both the cortex and striatum; however, EB extravasation significantly reduced in the first 1 h. Then, it gradually declined and returned to the same level as the control group approximately 6 h (cortex) and 4 h (striatum) after sonication. However, no evident changes of EB extravasation were found in the cortex and striatum of the control group at various time points after EB injection.

### Expression of TJ related proteins after BBB disruption

**Figure 9:**
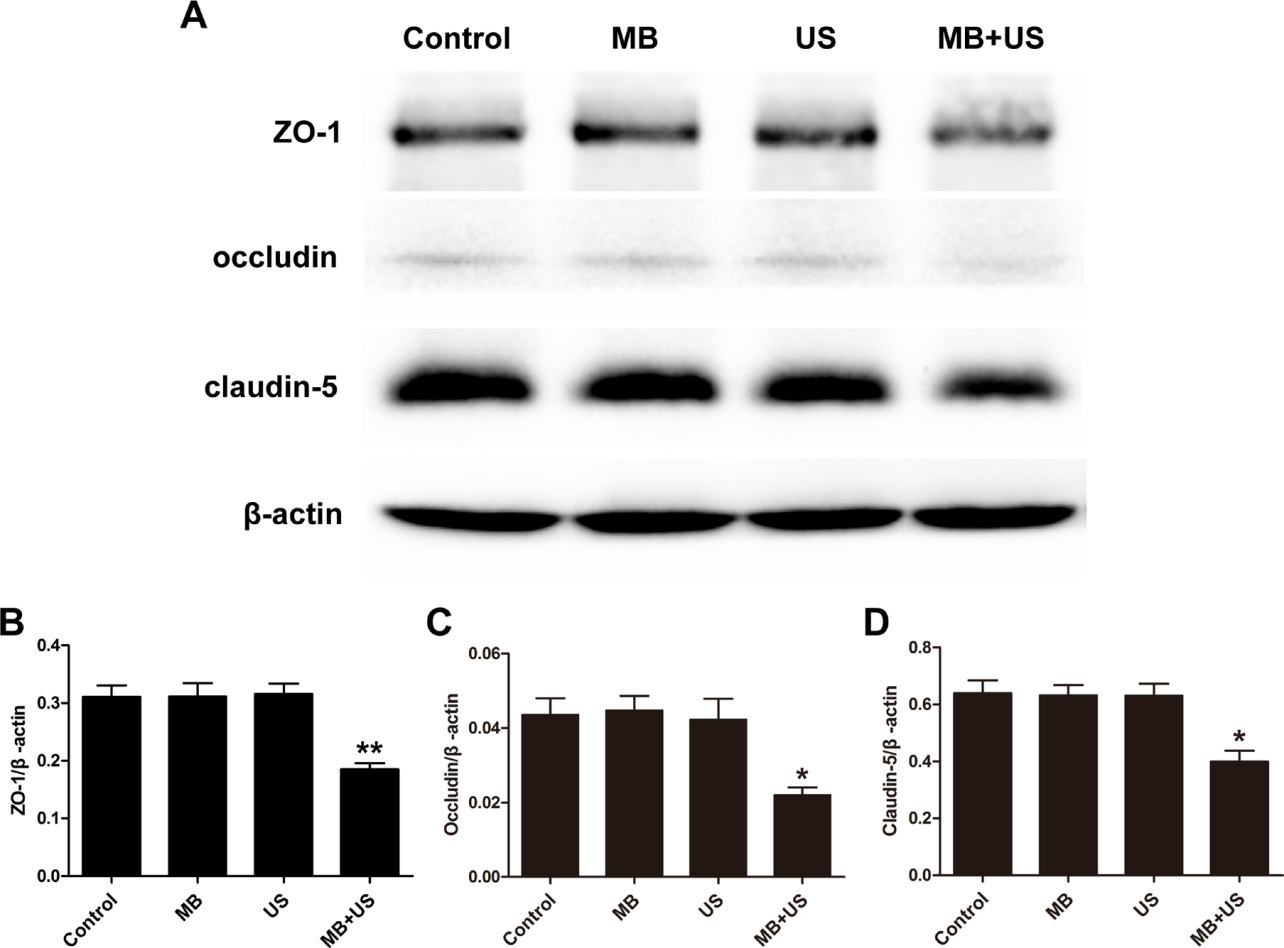
Representative blots (**A**) and relative quantitative analysis (**B**, **C**, **D**) of TJ related proteins ZO-1, occludin and claudin-5 expression in each group. Data were shown as the mean ± SEM, ^*^P < 0.05, ^**^P < 0.01 vs. control group, MBs only group and ultrasound only group. Control, without sonication or MB injections; MB, only MB injections and without sonication; US, only sonication and without MB injections.

**Figure 10:**
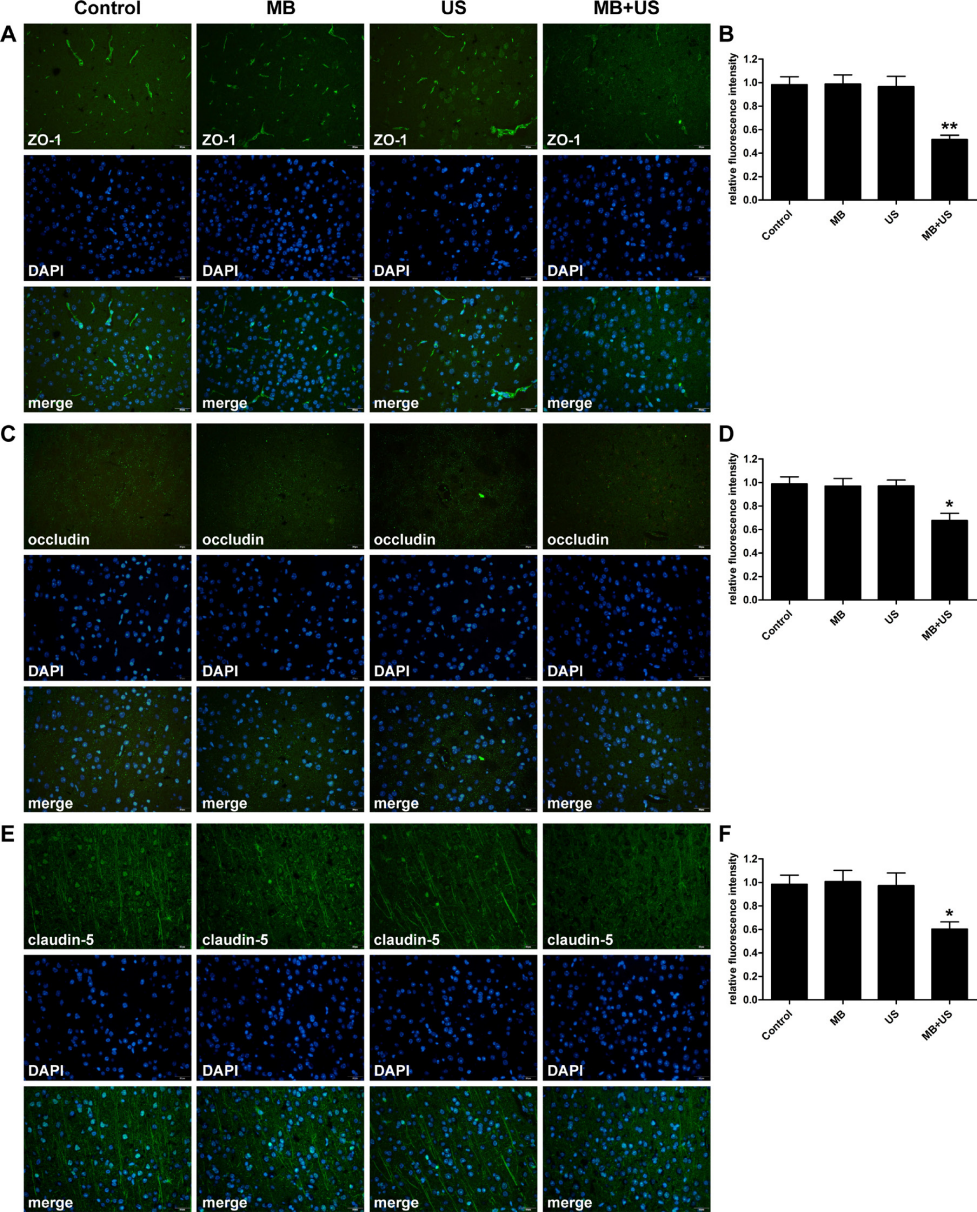
Distribution and expression level of TJ related proteins ZO-1 (**A**), occludin (**C**) and claudin-5 (**E**) observed via immunohistofluorescence staining in each group. Relative fluorescence intensity of ZO-1 (**B**), occludin (**D**) and claudin-5 (**F**) compare with the control group. Data were shown as the mean ± SEM, ^*^P < 0.05, ^**^P < 0.01 vs. control group. Control, without sonication or MB injections; MB, only MB injections and without sonication; US, only sonication and without MB injections. Scale bar = 20 μm.

To investigate the molecular mechanism of BBB disruption induced by MBs and ultrasound treatment, brain tissues were harvested, and the expression of TJ related proteins ZO-1, occludin and claudin-5 were subsequently analyzed by western blotting and immunohistofluorescence. As shown in Figure 9, compared with the control group, expression of ZO-1, occludin and claudin-5 significantly decreased after MBs and ultrasound treatment. However, there was no significant difference in the MBs only group and the ultrasound only group compared with the control group. In the immunohistofluorescence assay (Figure 10), positive cells of ZO-1, occludin and claudin-5 were significantly reduced after MBs and ultrasound treatment. However, there was no significant difference between the MBs only group, the ultrasound only group and the control group. These results were consistent with the results of western blotting.

### Ultrastructure changes of TJs after BBB disruption

**Figure 11:**
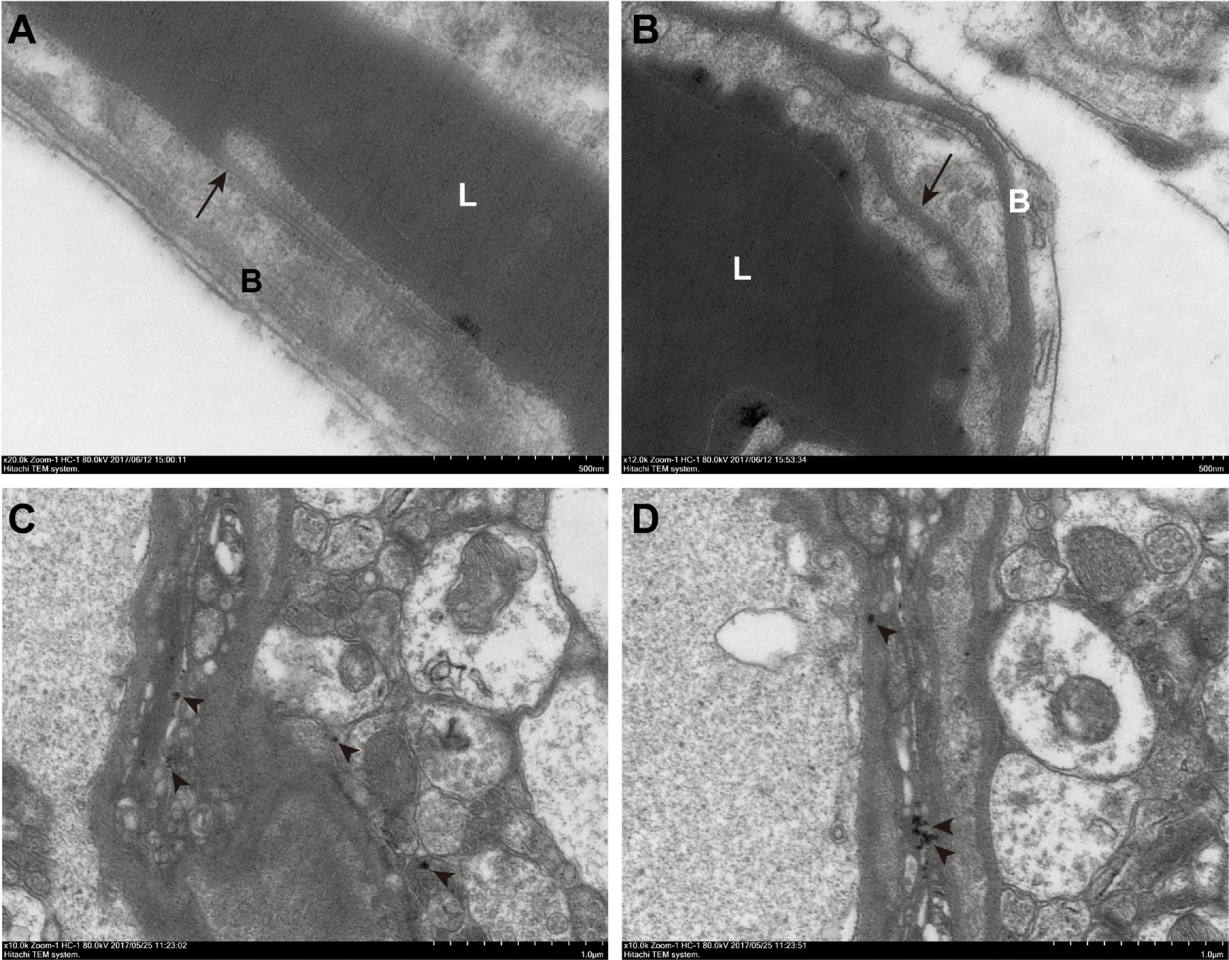
Transmission electrical microscopic observation of ultrastructure changes of TJs after BBB disruption (**A**) The control group. The tracer could only be seen on the luminal surface of endothelial cells (ECs), and ECs and basement membranes were free of lanthanum nitrate. (**B**–**D**) The group treated with MBs combined with ultrasound. The tracer passed through the entire interendothelial clefts (B, long arrow), deposited on the basement membrane (B), and penetrated deeply into the interstitial space of the surrounding neuropil (C, D, short arrow). Control, without sonication or MB injections. L, lumen; B, basement membrane; long arrow, tight junction; short arrow, lanthanum nitrate. Scale bar = 500 nm.

To observe ultrastructure changes of TJs after BBB disruption, lanthanum nitrate was applied as a tracer for BBB permeability. In the control group, no damage to vessel morphology was found. The tracer could only be seen on the luminal surface of ECs, and no tracer passed through the interendothelial clefts (Figure 11A). In contrast, in the mice treated with MBs combined with ultrasound, TJs of ECs were clearly disturbed, and lanthanum nitrate passed through the entire interendothelial clefts and deposited on the basement membrane (Figure 11B), even penetrating deeply into the interstitial space of the surrounding neuropil (Figure 11C and 11D).

## DISCUSSION

Numerous studies have demonstrated that FUS combined with MBs can locally and temporarily disrupt the BBB and enhance brain delivery of diagnostic and therapeutic agents with negligible side effects to the brain [20–25]. However, FUS is not suitable for the treatment of brain diseases with unclear or extensively distributed lesions because of limited focus spots. Moreover, the precise targeting of FUS was essentially achieved by the assistance of other methods such as an additional ultrasound imaging transducer, magnetic resonance imaging, a grid positioning method, etc [15, 20, 30]. Unfocused ultrasound generated by a diagnostic ultrasound system can overcome the aforementioned weakness of FUS. On one hand, without the limitation of focus spots, diagnostic ultrasound can induce a broader range of BBB disruption than FUS after one time ultrasound irradiation [26]. On the other hand, using the combination of imaging function of diagnostic ultrasound itself and the stereotaxic apparatus, we can pinpoint the internal localization of the brain more readily and efficiently. Therefore, in this study, a commercialized diagnostic ultrasound system was applied for BBB disruption, which was rarely used in previous studies. Effects of various experimental parameters (MB dose, MI and sonication duration) on the extent of BBB disruption under the flash mode were investigated. In addition, the potential molecular mechanism was preliminarily studied.

The relationship between MB dose and the extent of BBB disruption induced by diagnostic ultrasound was confirmed. Our results indicated that the volume and degree of EB staining increased with MB doses in the surface view and coronal sections, which also demonstrated by the quantitative analysis of EB extravasation. MBs consist of gas-filled cores and stabilizing phospholipids shells with diameters of 1–3 μm. The compressible property of the gas core allows MBs to undergo oscillation in response to each cycle of pressure within the ultrasound field [31]. Stable and inertial cavitation can generate a series of biological effects on the ECs, which can induce the disruption of the BBB [32]. A higher MB dose in the blood vessels provides more cavitation nuclei, thus lowering the energy required for cavitation, increasing the degree of BBB disruption, promoting EB extravasation and therefore appearing darker EB staining [33, 34].

MI, defined as the ratio of peak negative acoustic pressure (PNP, in MPa) to the square root of frequency (f, in MHz), has been demonstrated as a meaningful measurement to identify the threshold for BBB disruption induced by ultrasound combined with MBs [35]. At a low MI, MBs oscillate symmetrically near the vessel wall. The expansion of MBs can push ECs apart resulting in the opening of TJs via mechanical stretching. At a high MI, MBs oscillate violently and collapse rapidly, and micro-streams are created that can exert high shear stress on ECs to disrupt the BBB [36]. We found that the biological effect generated by the interaction between MBs and ultrasound may not be high enough to trigger BBB disruption at MI 0.2. However, when the MI increased to 0.4, EB extravasation significantly increased compared with the control. Thereafter, BBB permeability increased monotonically with increasing the MI. This result demonstrated that the threshold for BBB disruption might be approximately MI 0.4; the corresponding PNP was approximately 0.49 MPa, which was generally consistent with previous studies confirming that the threshold for BBB disruption ranged from 0.4 to 0.8 MPa, depending on the type of contrast agent, contrast agent dose and ultrasonic frequency [14, 35, 37].

Diagnostic ultrasound induced BBB disruption depends not only on MB dose and MI but also on sonication duration. Increasing sonication duration steadily increased the degree of EB staining and the amount of EB extravasation. Therefore, the extent of BBB disruption can be influenced by the application of appropriate sonication duration. It was additionally found that there was no visible EB staining in the MBs only group and the ultrasound only group, just like the control group, which once again showed that BBB disruption were induced by biological effects produced by the interaction between MBs and ultrasound. However, there was a small amount of EB extravasation detected in the MBs only group, the ultrasound only group and the control group, possibly attributable to the absence of an intact BBB in some brain structures such as the area postrema [38].

Heterogeneity of EB distribution in different regions of the brain was observed in this study, which related to tissue characteristics. The amount of EB extravasation per unit mass of striatum was higher than that of the cortex. Depth of a mouse brain is merely approximately 6 mm; therefore, the difference in acoustic pressure caused by ultrasound attenuation in the brain was negligible [39]. Uneven distribution of EB may be explained as the variable density of microvessels in different regions of the brain. Regions with higher microvessel density would have higher access to MBs to promote more EB extravasation. Therefore, diagnostic ultrasound induced BBB disruption can be used for the treatment of lesions in the striatum such as those found in Parkinson's disease patients.

Considering that an impermeable BBB is essential to maintaining CNS homeostasis, the safety of the technique may draw close attention. The possibility of adverse effects was evaluated by histological examination. There was no damage observed in the groups where the BBB was intact or very slightly disrupted, including the control group, the MBs only group, the ultrasound only group, as well as the groups sonicated with a MI less than or equal to 0.4. Increasing the MB dose to 1.0 × 10^7^ MBs, MI to 0.8, or sonication duration to 3 min, BBB disruption was only associated with a few scattered erythrocytes to small groups of erythrocyte extravasation. Nonetheless, it has been reported that erythrocyte extravasation had minimal effects on brain tissues, and such effects would be acceptable for the treatment of tumors and neurodegenerative diseases [40–42]. At a MB dose of 2.0 × 10^7^ MBs or a sonication duration of 4 min, erythrocyte extravasation increased and individual dark-stained ischemic or apoptotic neurons appeared. Although a MB dose of 3.0 × 10^7^ MBs can induce the largest extent of BBB disruption, it was accompanied by the most serious damage. Therefore, these findings indicate that BBB disruption with minimal tissue damage can be achieved by an appropriate MB dose and ultrasound parameters with diagnostic ultrasound.

In addition, we monitored the change of BBB permeability after BBB disruption under optimal parameters for 24 h. We found that the duration of BBB disruption in the cortex and striatum were no more than 6 h and 4 h, respectively. Duration of BBB disruption in the striatum was consistent with previous studies, while duration in the cortex was slight longer than their results (4 h) [26, 27]. One explanation would be that the duration of BBB disruption was related to the type of contrast agent, contrast agent dose and ultrasound exposure parameters. Therefore, this duration may suggest a window of opportunity for drug delivery into the CNS.

The highly impermeability of the BBB is primarily due to the existence of TJs, which consist of transmembrane proteins occludin, claudins (claudin-1 and claudin-5), submembranous zonula occludens proteins (ZO-1, ZO-2 and ZO-3) and the cytoskeleton of ECs [5]. Occludin determines tight junctional barrier and fence functionalities, while claudins contribute to paracellular ion and size selectivity [43]. Submembranous zonula occludens proteins are responsible for anchoring the transmembrane proteins to the cytoskeleton of ECs and signal transduction [44]. To explore the molecular mechanism of BBB disruption induced by diagnostic ultrasound combined with MBs, western blotting, immunohistofluorescence staining and transmission electron microscopy (TEM) analysis were performed. The results clearly indicated that the expression of all three TJ related proteins significantly decreased after MBs and ultrasound treatment, especially ZO-1. At the same time, the opening of TJs and the paracellular passage of the tracer lanthanum nitrate into the basement membrane and surrounding brain tissue were considered as morphological evidence of BBB disruption. We concluded that the decreased expression of TJ related proteins ZO-1, occludin and claudin-5 were associated with BBB disruption induced by diagnostic ultrasound combined with MBs. These finding are in accordance with the previous studies that BBB disrupted through osmotic insult or FUS [5, 45], suggesting that these proteins could be reliable and sensitive indicators of integrity of the BBB.

In this study, diagnostic ultrasound capable of real-time imaging of the relevant anatomical landmarks of the mice skull for precise targeting was applied to induce BBB disruption noninvasively in the presence of MBs. Extent of BBB disruption increased with MB dose, MI and sonication duration. Concurrently, the risk of tissue damage additionally increased. A relatively larger extent of BBB disruption associated with minimal tissue damage could be achieved by an appropriate MB dose and ultrasound exposure parameters with diagnostic ultrasound. In addition, under optimal parameters, disruption of the BBB was reversible allowing recovery within 6 h and 4 h in the cortex and striatum, respectively. Reduced expression of TJ related proteins ZO-1, occludin and claudin-5 were correlated with disruption of the BBB, which was determined by paracellular passage of the tracer lanthanum nitrate into the basement membrane and surrounding brain tissue using TEM. These findings together indicated that diagnostic ultrasound with the aid of MBs could enhance the permeability of the BBB effectively and noninvasively and might serve as a promising tool for brain delivery of diagnostic and therapeutic agents for brain diseases.

## MATERIALS AND METHODS

### Preparation of MBs

MBs used in this experiment were prepared by the thin-film hydration method. Briefly, 1,2-distearoyl-sn-glycero-3-phosphatidylcholine (DSPC, CordenPharma Switzerland LLC, Liestal, Switzerland) and 1, 2- distearoyl-sn-glycero-3-phosphoethanolamine-N- [methoxy (polyethylene glycol)-2000] (DSPE-PEG-2000, CordenPharma Switzerland LLC, Liestal, Switzerland) were dissolved in chloroform at a molar ratio of 9:1. The chloroform was removed by evaporation under a steady nitrogen stream at room temperature until a thin lipid film formed, followed by drying in a vacuum over 2 h. The lipid film was hydrated with a solution of 10:10:80 (v/v/v) glycerol solution: propylene glycol: 0.1 M Tris-buffered saline (pH 7.4) at 60°C, and sub-packaged into vials (1 ml each vial). Gas in each vial was removed and refilled with perfluoropane gas (C_3_F_8_). After mechanical shaking via an agitator for 30 s, MBs with a lipid shell and a C_3_F_8_ gas core were formed. Free lipids were removed by centrifuging at 700 rpm for 3 min. Morphology of MBs were observed under bright-field microscopy and fluorescent microscopy after being stained by DiI (Beyotime Biotechnology, Shanghai, China). Size distribution and concentration of MBs were measured by a dynamic light-scattering system (ZetaPALS Zeta Potential Analyzer, Brookhaven Instruments Corp, Holtsville, NY, USA).

### Experimental animals

All procedures for animal experiments were approved by the Institutional Animal Care and Use Committee of Tongji Medical College, Huazhong University of Science and Technology, and performed in accordance with the experimental animal care guidelines. Male C57-BL6 wild type mice (20–25 g) were provided by the Hubei province experimental animal research center (Wuhan, China). Animals were housed in a specific pathogen free (SPF) environment on a 12 h light-dark cycle with access to food and water ad libitum.

### Ultrasound system

A commercialized Vivid E9 diagnostic ultrasound system (GE Healthcare, Milwaukee, WI, USA) was used in this study. An ultrasound beam was generated by a M5S-D phased array transducer operating in the second harmonic mode (transmit: 1.5 MHz, receive: 3.0 MHz). The transducer was positioned using a stereotaxic apparatus (RWD Life Science Co., Ltd, Shenzhen, China) to ensure the acoustic beam targeted the brain precisely. The transducer was submersed in a water tank containing deionized and degassed water whose bottom was sealed by a polyurethane membrane. Focal depth was set at 5 cm, which is approximately 3 mm below the dorsal surface of the skull.

### BBB disruption procedure

**Figure 12:**
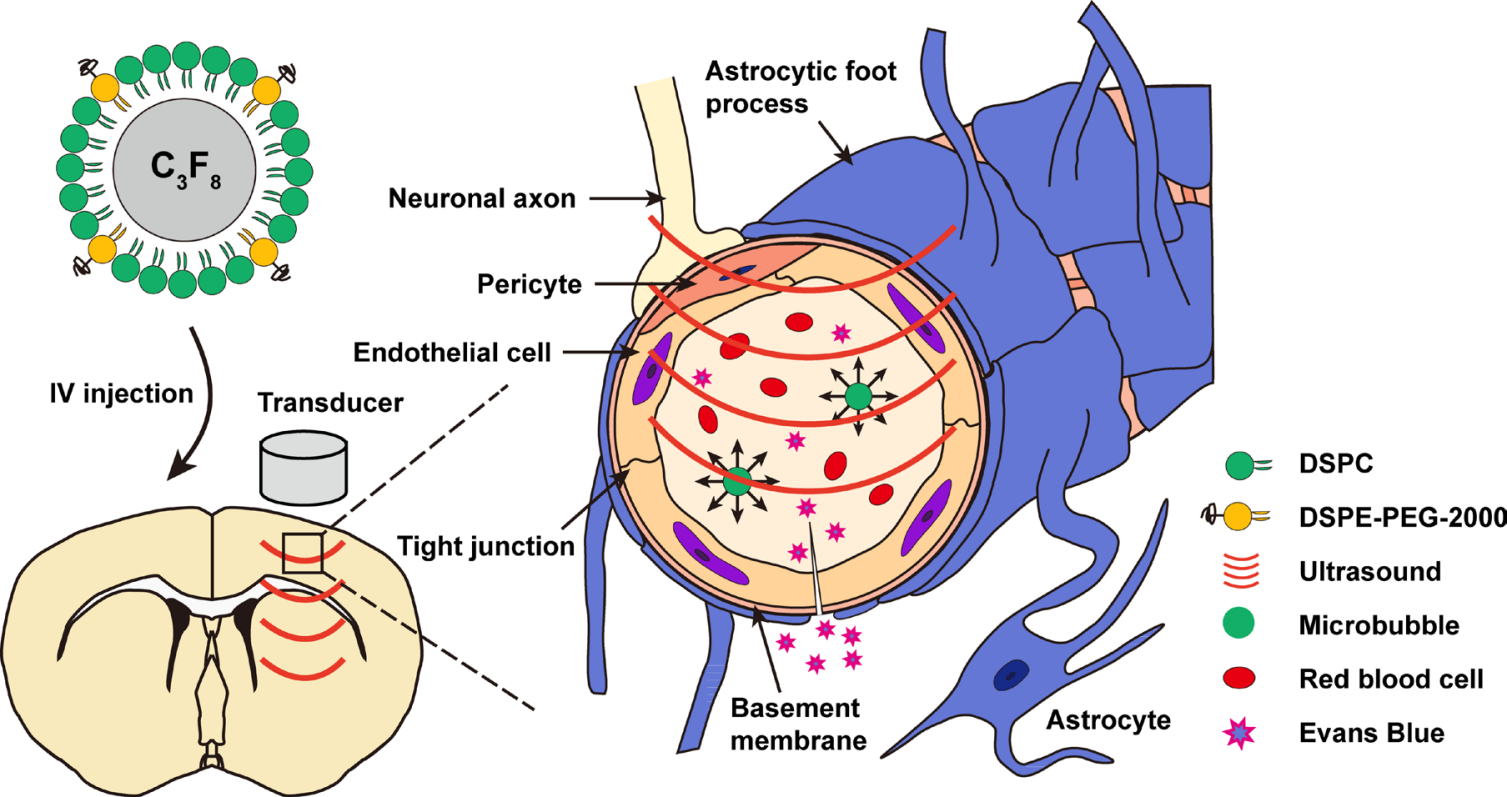
Schematic representation of BBB disruption induced by diagnostic ultrasound in combination with intravenous MB injections IV, intravenous.

Prior to the experiment, animals were anesthetized intraperitoneally with chloral hydrate (300 mg/kg). Mouse hair over the skull was removed using an electric trimmer and depilatory cream. For sonication, the head of the mouse was immobilized by a stereotaxic apparatus in a prone position beneath the water tank, and ear bars and a bit bar were adjusted to make the dorsal skull surface horizontal. Ultrasound coupling gel was applied between the polyurethane membrane and the scalp to maximize transmission of the ultrasound. Body temperature of the animals was maintained at 36.5 ± 0.5°C using a heating blanket during the experiment. With real-time guidance of ultrasound images and the aid of a stereotaxic apparatus, sonication was delivered to the right striatum region at the position of 3 mm posterior to the right eye and 2 mm lateral to the midline. A bolus of MBs was intravenously injected via the tail vein approximately 15 s before sonication (Figure 12).

Three experimental protocols were performed (Table 1). In the first protocol for optimizing parameters, the degree of BBB disruption influenced by various MB doses (0.5 × 10^7^ MBs, 1.0 × 10^7^ MBs, 2.0 × 10^7^ MBs and 3.0 × 10^7^ MBs in 30 μl saline solution), MIs (0.2, 0.4, 0.6 and 0.8) and sonication durations (1 min, 2 min, 3 min and 4 min) at the same region was evaluated. Three additional groups of animals served as controls: the first group received no sonication or MB injections (denoted as the control group), the second group received sonication without MB injections (ultrasound only group), and the third group received MB injections without sonication (MBs only group). EB (Sigma-Aldrich, St. Louis, MO, USA) was injected intravenously at a dose of 100 mg/kg immediately after sonication. Histological examination was applied to assess potential tissue damage. In the second protocol, mice were injected intravenously with EB at various preset time points (0 h, 0.5 h, 1 h, 2 h, 4 h, 6 h, 24 h) after sonication under the optimal parameters to evaluate the duration of BBB disruption. In the third protocol, expression of TJ related proteins ZO-1, occludin and claudin-5 was examined by western blotting analysis and immunohistofluorescence staining of the control group, MBs only group, ultrasound only group and MBs combined with ultrasound group. TEM was performed to observe ultrastructure changes of TJs after BBB disruption.

**Table 1:** Experimental parameters for different groups of animals in this study

Group	MB dose	MI	Sonication duration	EB injection time
First protocol	-	-	-	Immediately injection
	-	0.8	3min	Immediately after sonication
	1.0 × 10^7^	-	-	Immediately after MB injections
	(0.5–3.0) × 10^7^	0.8	3 min	Immediately after sonication
	1.0 × 10^7^	0.2–0.8	3 min	Immediately after sonication
	1.0 × 10^7^	0.8	1–4 min	Immediately after sonication
Second protocol	1.0 × 10^7^	0.8	3 min	0–24 h after sonication
Third protocol	-	-	-	Immediately injection
	-	0.8	3 min	5 min prior to sonication
	1.0 × 10^7^	-	-	5 min prior to MB injections
	1.0 × 10^7^	0.8	3 min	5 min prior to sonication

MB, microbubble; MI, mechanical index; EB, Evans Blue.

### Assessment of BBB integrity

To evaluate the influence of MB dose, MI and sonication duration on BBB integrity, animals were sacrificed approximately 6 h after the EB injection. Mice were deeply anaesthetized with chloral hydrate and infused with heparinized saline via the left ventricle until a colorless infusion liquid was obtained from the right atrium; subsequently, brains were removed. The brains were sliced into six 1.5-mm-thick coronal sections. First, a qualitative analysis examined the degree and volume of EB extravasation in the brain to characterize BBB disruption. Second, a quantitative analysis measured the amount of EB extravasation in the cortical and striatum of the right hemisphere. Brain tissue samples were weighted separately and placed in 50% trichloroacetic acid solution. After homogenization, the mixture was centrifuged for 20 min at 12000 rpm. Supernatant was diluted with absolute ethyl alcohol (1:3). Fluorescence intensity was measured at 680 nm using a fluorescence spectrophotometer with excitation at 620 nm (PerkinElmer, LS55, UK). The amount of EB extravasation in each brain tissue sample was calculated by a linear regression standard curve obtained from a serial dilution of EB standard solution and was expressed as the amount of EB per gram of brain tissue sample.

### Histological examination

Six hours after the injection of EB, animals were anesthetized with an overdose of chloral hydrate and successively infused with heparinized saline and 4% paraformaldehyde. Brains were then removed, immersed in 4% paraformaldehyde for 24 h, and dehydrated with graded ethanol solutions. Brain tissue samples taken from sonicated sites were embedded in paraffin, which were easily identified by EB, and serially sectioned at 4 μm thickness in the coronal plane (parallel to the direction of ultrasound beam propagation). Every 50th section was subjected to hematoxylin and eosin (H&E) staining for histological examination to evaluate erythrocyte extravasation and other tissue damage caused by the technique.

### Duration of BBB disruption

To investigate the duration of BBB disruption, permeability of the BBB was assessed through quantitative analysis of the amount of EB extravasation injected at various specific time points (0 h, 0.5 h, 1 h, 2 h, 4 h, 6 h and 24 h) after sonication. Approximately 6 h after the EB injection, animals were sacrificed and the amount of EB extravasation was measured as mentioned above.

### Western blotting analysis

After undergoing the third protocol described above, mice were sacrificed immediately. Brains were removed, and sonicated tissues and corresponding tissues in the three other control groups were isolated rapidly. All proteins were extracted using RIPA lysis buffer containing PMSF and a protease inhibitor cocktail. Protein concentration was determined by a bicinchoninic acid (BCA) assay (Beyotime Biotechnology, Shanghai, China). Equal amounts of protein (50 μg) were separated by 6% or 12% SDS-PAGE gels and transferred onto PVDF membranes. Membranes were blocked in 5% non-fat milk in Tris-buffered saline plus 0.1% Tween-20 (TBST) for 2 h at room temperature. Membranes were incubated overnight at 4°C with a rabbit polyclonal antibody to ZO-1 (1:1000, Invitrogen, Thermo Fisher Scientific, Waltham, MA, USA), a rabbit polyclonal antibody to occludin (1: 500, Abcam, Cambridge, MA, USA), a rabbit polyclonal antibody to claudin-5 (1: 1000, Abcam, Cambridge, MA, USA) and a mouse monoclonal antibody to β-actin (1:1000, Beyotime Biotechnology, Shanghai, China). Then, secondary HPR-conjugated goat antibodies against rabbit or mouse (Beyotime Biotechnology, Shanghai, China) were applied at a dilution of 1:1000 for 2 h at room temperature. Protein bands were detected by an enhanced chemiluminescence kit (Beyotime Biotechnology, Shanghai, China) using ChemiDoc XRS+ (Bio-Rad Laboratories, Hercules, CA, USA), and relative protein amount was analyzed using Image J software (National Institutes of Health, Bethesda, MD, USA).

### Immunohistofluorescence staining

Mice were killed immediately after treated according to the third protocol with their brains fixed, dehydrated, embedded in paraffin and sliced as described above. After deparaffinization, hydration and antigen retrieval, sections were blocked with 3% BSA for 30 min at room temperature, then incubated overnight at 4°C with the following primary antibodies: rabbit anti-ZO-1 (1:100, Invitrogen, Thermo Fisher Scientific, Waltham, MA, USA), rabbit anti-occludin (1: 100, Abcam, Cambridge, MA, USA) and rabbit anti-claudin-5 (1: 100, Abcam, Cambridge, MA, USA). Sections were rinsed three times in PBS, incubated for 1 h at room temperature with Alexa Fluor^®^ 488-conjugated goat anti-rabbit IgG (1: 1000, Abcam, Cambridge, MA, USA), and stained with 4′,6-diamidino-2-phenylindole (DAPI) for 10 min. Images were acquired under an inverted fluorescence microscope (Olympus IX71, Olympus Optical Co., Ltd, Tokyo, Japan).

### Transmission electron microscopy analysis

Immediately after sonication, animals were re-anesthetized and transcardially infused first with a saline solution containing 10 mM lanthanum nitrate (Sigma-Aldrich, St. Louis, MO, USA), followed by infusion with 4% paraformaldehyde and 1% glutaraldehyde in 0.1 M PBS (PH 7.4) and then 2% lanthanum nitrate solution. With brains removed, tissue blocks of approximately 1 mm^3^ obtained from sonicated sites and corresponding areas in the control group were fixed with 2.5% glutaraldehyde for 2 h at 4°C, washed in PBS, postfixed in 1% osmium tetraoxide for 2 h, dehydrated in ethanol, and embedded in Epon 812. Ultrathin sections stained with uranyl acetate and lead citrate were examined by TEM (Hitachi HT7700, Hitachi Ltd., Tokyo, Japan) at an accelerating voltage of 80 kV.

### Statistical analysis

Statistical analysis was performed using GraphPad Prism 5.01 software (GraphPad Software, Inc., San Diego, CA, USA). All data are present as the mean ± standard error of the mean (SEM) for each group. Unpaired student's *t*-tests were used for comparisons between two groups, while one-way analysis of variance (ANOVA) with the Bonferroni post hoc test was applied for multiple comparisons. Statistical significance was defined as *P* values < 0.05.

## Abbreviations

BBB: blood-brain barrier
CNS: central nervous system
ECs: endothelial cells
TJ: tight junction
FUS: focused ultrasound
MB: microbubble
MI: mechanical index
DiI: 1:1′-dioctadecyl-3:3:3′:3′-tetramethylindocarbocyanine perchlorate
EB: Evans Blue
H&E: hematoxylin and eosin
PNP: peak negative acoustic pressure
DSPC: 1:2-distearoyl-sn-glycero-3-phosphatidylcholine
DSPE-PEG-2000: 1:2-distearoyl-sn-glycero-3-phosphoethanolamine-N-[methoxy (polyethylene glycol)-2000]
TEM: transmission electron microscopy
SPF: specific pathogen free
IV: intravenous
BCA: bicinchoninic acid
TBST: Tris-buffered saline plus 0.1%Tween-20
SEM: standard error of the mean
ANOVA: one-way analysis of variance
